# How to Solve the Social Norm Conflict Dilemma of Green Consumption: The Moderating Effect of Self-Affirmation

**DOI:** 10.3389/fpsyg.2020.566571

**Published:** 2020-11-24

**Authors:** Wanda Ge, Guanghua Sheng, Hongli Zhang

**Affiliations:** ^1^School of Management, Qilu University of Technology (Shandong Academy of Sciences), Jinan, China; ^2^School of Business, Jilin University, Changchun, China

**Keywords:** green consumption, social norm conflict, alienation, powerlessness, meaninglessness, self-affirmation

## Abstract

Social norms are important social factors that affect individual behavioral change. Using social norms to promote green consumption is receiving increasing attention. However, due to the different formation processes and mechanisms of the behavioral influence of the different types of social norms, using social norms to promote green consumption often has social norm conflict situations (injunctive norms + negative descriptive norms). Thus, it is difficult to attain the maximum utility of social norms. The present research found that social norm conflict weakens the role of injunctive norms in promoting green consumption. Specifically, negative descriptive norms weaken the role of injunctive norms in promoting green consumption. Alienation, which manifests through powerlessness and meaninglessness, plays a mediating role in the relationship between social norm conflict and green consumption. Self-affirmation moderates the mediating role of alienation between social norm conflicts and green consumption. Self-affirmation reduces the alienation caused by social norm conflict, thereby alleviating the weakening effect of social norm conflict on green consumption.

## Introduction

Green consumption, also known as sustainable consumption, refers to consumption behavior wherein consumers consider the environmental impact when buying, using, or disposing products to minimize environmental harm (Carlson et al., 1993; Roozen and De Pelsmacker, 2000; Wang et al., 2019). On the one hand, environmental degradation is largely caused by residents’ unreasonable consumption habits and consumption patterns, and green consumption is crucial in solving environmental problems (Smith et al., 2012; Sheng et al., 2019). On the other hand, green consumption can form a green market demand to protect the ecological environment, forcing enterprises to formulate green development strategies and leading enterprises to adopt green environmental protection (Sheng et al., 2017; Pongpiachan, 2019; Nguyen et al., 2019). Green consumption requires consumers to increase their efforts by, for instance, paying environmental premiums for green products and changing original consumption habits. The environmental benefits generated by green consumption are not only individually enjoyed by consumers but also shared by the whole society. The generation of green consumption behavior requires consumers to balance their interests with social interests (Olson, 2013). The generation and solution of environmental problems are of a natural collective character and require the collective participation of all members of society (Fritsche et al., 2018). Therefore, even if consumers realize the importance of protecting the environment, “high green consumption preference” and “low green consumption behavior” may occur. Focusing on effectively realizing green consumption, scholars have conducted a series of useful explorations. Among them, social norms have become the focus of scholars’ attention in recent years (Eom et al., 2016; Sparkman and Walton, 2017; Fritsche et al., 2018; Pongpiachan, 2018).

Social norms are behavioral standards gradually formed in social practice and social interaction to regulate individual behaviors to maintain social order (Magill, 1995; Nolan et al., 2008), and these can be divided into descriptive and injunctive norms (Cialdini et al., 1990; Farrow et al., 2017). Descriptive norms are behavioral standards formed by the behaviors of most group members (Cialdini et al., 1990; Farrow et al., 2017), such as “75% of consumers have already made green consumption.” Descriptive norms can reflect positive (respected) or negative (prohibited) behaviors. Injunctive norms are standards of conduct formed by the majority of members of a group in favor of or against a certain behavior (Farrow et al., 2017). This includes codes of conduct issued by the government or a spontaneously formed within a group. An example of this could be: “people highly agree with green consumption.” Compared with commonly used arguments are employed in public appeals to encourage green consumption (such as “protecting the environment, everyone is responsible”), social norms have received increased attention from scholars due to their low implementation cost and effective intervention effect, and social norms have been applied into practice. However, social norm conflicts also occur in real life (McDonald et al., 2013; Chen and Hong, 2015). Social norm conflict refers to the situation in which injunctive norms and negative descriptive norms coexist (injunctive norms + negative descriptive norms). Sometimes social norm conflicts are demotivating and undermining actions (Schultz et al., 2007; McDonald et al., 2014; Hassell and Wyler, 2018). The effects of social norm conflict are likely to be relevant to conservation or saving behaviors, particularly where the behavior in question is visible and must be enacted collectively (McDonald et al., 2014).

As green consumption needs customers to distribute various costs, and the environmental benefits generated by green consumption are uncertain, negative descriptive norms will arise and may discourage many to refrain from conducting green consumption (Sheng and Ge, 2019). If the injunctive norm is used to promote green consumption, a social norm conflict has occurred. Negative descriptive and injunctive norms coexist, and injunctive norms cannot effectively promote green consumption. The focus theory of normative conduct believes that the situation of conflicting social norms will make negative descriptive norms the focus of consumers’ attention. Currently, consumers will preferentially follow negative descriptive norms, making it difficult for social norms to play a regulatory role in consumer behavior (Cialdini et al., 1990). However, few studies have focused on the issue of conflicting social norms of green consumption (injunctive norms + negative descriptive norms) nor have they explored the internal psychological process of conflicts of social norms affecting green consumption or found effective solutions to resolve the negative impact of conflicts of social norms on green consumption.

This paper contributes to the literature in several ways. First, based on the social norm conflict in green consumption and the focus theory of normative conduct, this study explores the negative impact of social norm conflict on green consumption. Second, the concept of alienation is introduced to explain the internal influence mechanism between social norm conflict and green consumption. Third, we examine how self-affirmation can be used to alleviate the negative impact of social norm conflict on green consumption based on the self-integrity theory.

## Theoretical Review and Research Hypothesis

### Social Norms and Conflicts of Social Norm

Social norms refer to the rules and standards that guide or limit the behavior of group members to ensure the realization of group goals and consistency of group activities (Cialdini et al., 1990; Cialdini and Goldstein, 2004). Different from the mandatory legal system, social norms are based on informal social sanctions or rewards, which guarantee the implementation of behavior. American psychologist Cialdini et al. (1990) proposed the focus theory of normative conduct, dividing social norms into descriptive and injunctive norms. Descriptive norms tell people what kind of behavior is effective, acceptable, and safest in a specific situation, which is related to effective personal goals and more likely stimulates the individual’s heuristic information-processing mode. When individuals face descriptive norms, they need not spend a lot of cognitive resources to think about their behaviors. Descriptive norms help individuals make quick decisions (Cialdini et al., 1990; Jacobson et al., 2011). The effects of descriptive norms on behavior are similar to the generation of herd behavior. The safest and most effective practices are those of the majority, and individuals tend to emulate this behavior (Cialdini et al., 1990; Cialdini and Goldstein, 2004; Zhang, 2016). Injunctive norms reflect the value judgment tendency of most members of the group and guide people’s behavior by emphasizing the good or bad behavior of the group, stimulating the individual’s systemic information-processing mode. When individuals are facing injunctive norms, they think systematically about the content of the norms. Considering the appropriateness of their behaviors, group members will expend more cognitive resources (Cialdini et al., 1990; Cialdini and Goldstein, 2004; Stok et al., 2014a). Only when the majority of the members of the group approve or disapprove a certain behavior will the injunctive norm have effect, and the value judgment of certain behavior by a few members will neither form a social norm nor will a “social sanction force” that restricts behaviors (Zhang, 2016).

Studies have shown that consumers considering themselves engaged in green consumption are still driven by many internal psychological factors, such as environmental values, environmental attitudes, and other moral characteristics. However, results showed that normative messages can be quite persuasive, and that their influence is often overlooked and underdetected (Nolan et al., 2008; Sheng and Ge, 2019; Eyink et al., 2020). Due to the effective normative effect on behaviors, social norms have been used to guide green consumption behaviors in the stages of a green product purchase, product use, and product recycling. For example, Schultz (1999) fed back social norms and advocacy information about garbage collection to residents’ families. Schultz found that the amount of garbage collection in those families that received social normative information was significantly higher than those families that were fed advocacy information. Moreover, through experimental research, Sheng and Ge (2019) found that compared with ordinary environmental protection information, injunctive normative information can significantly enhance consumers’ social value perception of green products, thus enhancing their purchase of green products. However, the forming process of descriptive and injunctive norms and their impact on behavior is different. Further, different types and directions of social norms may exist simultaneously, resulting in conflicts of social norms (Chen and Hong, 2015). Keizer et al. (2011) believed that a dirty and messy environment will highlight the negative descriptive norm of “everyone litters,” at which point individuals tend to litter. If the situation is accompanied by an injunctive norm (such as “no littering”), littering behavior may increase. At this point, the descriptive norm is not consistent with the injunctive norm, and social norms react against the guidance of behavior (Keizer et al., 2008).

Injunctive norms reflect generally accepted behavior in society and are the correct code of conduct; descriptive norms reflect behaviors generally exhibited by others and may be either consistent with injunctive norms or inconsistent with injunctive norms. Descriptive norms have both positive and negative directions (Smith et al., 2012; Chen and Hong, 2015). Positive descriptive norms are those in which most members of the group exhibit behavior consistent with injunctive norms, and there are no norm conflicts; negative descriptive norms mean that most members of the group exhibit behaviors inconsistent with injunctive norms. When injunctive norms are used to guide people’s behavior, social norm conflicts occur (injunctive norms + negative descriptive norms). In the case of norm conflict, the focus theory of normative conduct states that individuals will first follow descriptive norms (Cialdini et al., 1990; Keizer et al., 2008) because descriptive norms can provide a smooth information-processing process for individuals, and individuals will make behavioral decisions based on this reference point (Stok et al., 2014a; Zhang, 2016; Farrow et al., 2017). Thus, descriptive norms will now be the focus of individual attention, and individuals believe at this point that it is the best behavior in accordance with descriptive norms. Many social norm conflicts exist in real life, and an example of this is “Chinese-style road crossing.”

### The Weakening Impact of Social Norm Conflict on Green Consumption

For green consumption, using injunctive norms alone can positively enhance consumers’ willingness to implement green consumption in theory. Injunctive norms can associate green consumption with the attitudes of most members of the group. Group members will recognize green consumption behaviors that obey the injunctive norms and deny behaviors that do not comply with these norms and apply social sanctions (Stok et al., 2014b; Chen and Hong, 2015). In addition, injunctive norms can also enable individuals to realize normative behaviors in the context of social approval or disapproval of normative behavior, reevaluate the importance and rationality of this behavior, and comply with the injunctive norms (Paek et al., 2014; Farrow et al., 2017).

However, when injunctive norms are used to guide consumers’ green consumption behavior, social norm conflicts will occur (injunctive norms + negative descriptive norms). Since green consumption incurs costs on the consumers (Olson, 2013), most consumers cannot consciously practice green consumption (Yue et al., 2020). Thus, negative descriptive norms arise, and negative descriptive and injunctive norms will generate conflict. For consumers influenced by eastern culture, they have a high sense of group consciousness and are more often influenced by group opinions. The conflict situation of social norms encourages them to abide by negative descriptive norms (Keizer et al., 2008; Wang et al., 2016). Aiming at the dilemma of conflicting social norms in green consumption, this study believes that environmental improvement benefits brought by green consumption require the cooperation of all members of society. Injunctive norms will make consumers aware of the urgency of implementing green consumption when other consumers do not consciously implement the descriptive norms of green consumption. However, consumers will also believe that their green consumption behavior will not have much impact on the improvement of the environment as group pressure is absent. This leads consumers to relax their self-control in their consumption behavior. They tend to show behavior consistent with negative descriptive norms, that is, tend not to conduct green consumption. Therefore, the following hypotheses are proposed in this study:

H1: Social norm conflicts weaken the role of injunctive norms in promoting green consumption. Specifically, negative descriptive norms will weaken injunctive norms in promoting green consumption.

### The Mediating Effect of Alienation

Alienation was originally a philosophical proposition, referring to a situation wherein an individual is separated from the existing world. In sociology, alienation, a negative feeling of being estranged from others or the existing world, is a pervasive theme (Tome et al., 2016). For instance, when immigrants reject or dissociate from prevailing social norms and values, they may face cultural alienation (Miller et al., 2009). When workers realize that their work situation cannot meet their needs or is inconsistent with their expectations, individuals may feel alienated from work. This is when alienation becomes a psychological state that separates him from the job (Banai et al., 2004). When adolescents lack functional ties with primary socialization agents, including family and school, he may have a feeling of adolescent alienation (Slater, 2010). Although alienation can be defined in several ways, Seeman’s (1959) original conceptualization (five characteristics: powerlessness, meaninglessness, normlessness, isolation, and self-estrangement) referred to a more general sense of alienation from mainstream society’s values. Negative outcomes of alienation include feelings of despair, hopelessness, stress, anxiety, anguish, tension, or demoralization. Furthermore, alienation can lead to bad things. Immigrants separated from the local context will be at high risk to acculturative stress and isolation (Miller et al., 2009). Work alienation usually manifests externally as counterproductive work behavior, such as job burnout, lack of enthusiasm, and decreased performance (Li and Chen, 2018). Parent–teen alienation and higher rates of delinquent behavior and psychosocial maladjustment are correlated (Clarke et al., 2020).

Negative descriptive norms weaken the role of injunctive norms in promoting green consumption. To explain this weakening effect, this study suggests that alienation can provide a unique perspective. Alienation is often used as a sensitive and effective mediator to explain employees’ negative behavior (Ceylan and Sulu, 2010). We consider two main dimensions of alienation (powerlessness and meaninglessness) in this study. Previous research has shown that social norm conflicts affect their perceptions of the effectiveness of performing environmental behavior. Green consumption can provide long-term benefits for human beings, but generating long-term benefits also requires everyone’s participation (Fritsche et al., 2018). Norm conflict signals that not all others are acting and taking action are therefore ineffective and futile (McDonald et al., 2014). Therefore, social norm conflict of green consumption will lead to alienation between consumers and green consumption, specifically, powerlessness and meaninglessness. Powerlessness means that consumers believe their efforts have little impact and cannot help achieve the purpose of improving the environment. Meaninglessness implies that consumers cannot feel the environmental value and significance of their practice of green consumption. Therefore, the following hypotheses are proposed in this study:

H2: Alienation mediates the weakening effect of social norm conflict on green consumption.
H2a: Powerlessness mediates the weakening effect of social norm conflict on green consumption.
H2b: Meaninglessness mediates the weakening effect of social norm conflict on green consumption.

### The Moderating Effect of Self-Affirmation

Individuals are generally motivated to maintain a degree of self-integrity. Self-integrity is the individual’s belief that he (she) is generally good, moral, and fit for society (Steele, 1988). When self-integrity is threatened, psychological threats arise, and individuals can maintain their moral identity by thinking about their self-value in areas unrelated to threats or engaging in other activities of self-value through self-affirmation (Steele, 1988; Lv and Zhao, 2017). In other words, individuals can maintain self-integrity by compensating for their shortcomings in some areas with their strengths in other areas (Sherman and Cohen, 2006). In this process, because the individual’s self-value is anchored, they can process and accept threat information in a more open, fair, and objective manner. This weakens threatening information, which is the basic idea of the self-affirmation theory (Steele, 1988). As self-affirming individuals maintain their integrity, they can make significant changes in their cognitive, emotional, and behavioral tendencies and beneficial effects, such as objectivity, emotional relief, improved self-control, and growth promotion (Shi and Liu, 2009). Self-affirmation can reduce the individual’s defensive processing of threat information, making it easier for individuals to encode and extract threat information and improve information quality, and individuals can integrate threat information with themselves better, which will lead to adaptive behavior (van Koningsbruggen et al., 2009). Self-affirmation can positively enhance an individual’s positive implicit emotion so individuals can objectively assess self-threatening information (Crocker et al., 2008). Self-affirmation can encourage individuals to focus on the abstract, core, and essential characteristics of information and long-term goals and significance of their behaviors and improve the level of individual self-control (Schmeichel and Vohs, 2009). Because of the many beneficial effects of self-affirmation, Harris et al. (2017) believe that the reason is that self-affirmation affects the individual’s potential ability “executive functioning.” Since self-affirmation maintains one’s integrity, and self-integrity is a necessary condition for individuals to stay focused, set and complete goals, and to reason, self-affirmation indirectly improves the individual’s inhibitory control ability and reduces working memory tasks, therefore it leads to beneficial effects.

As mentioned, social norm conflict will weaken the promotion effect of injunctive norms on green consumption. Mitigating this weakening effect has become an urgent issue that needs to be addressed. This study suggests that self-affirmation can mitigate this weakening effect. Social norm conflict can make negative descriptive norms become the focus of consumers’ attention. The non-objectivity of this information-processing mode makes consumers pay too much attention to others’ “non-action” on green consumption and refuse to conduct green consumption on the grounds that others do not engage in this activity. Self-affirmation can maintain individual integrity, promote the openness of individual information processing, and enable individuals to assess information more objectively, fairly, and calmly (Cohen et al., 2007). Sherman and Hartson (2011) also proposed a three-stage model to explain the objective processing effect of self-affirmation on information. Self-affirmation is believed to first enhance self-resources. Second, individuals with self-resources, rather than simply accepting information, can respond to information with a broader perspective. Eventually, individuals will pay less attention to threat information and isolate it. After self-affirmation, consumers can effectively distinguish between short-term satisfaction and long-term interests and gain more overall awareness. They can effectively control their impulses and maintain self-control (He and Huang, 2012). Self-affirmation enables individuals to judge related events with more adherence to the ultimate purpose (Wakslak and Trope, 2009). This study believes that self-affirmed individuals will deal with conflict information more calmly and objectively when confronted with the social norm conflicts on green consumption. First, these individuals control their impulses, automatically alienate negative descriptive norms, and only focus on the injunctive norms that transmit positive social values. Second, this manifests in the state of construal level, and, in facing the conflicts of social norms, they think more on the positive results brought by consuming green products and focus on the environmental benefits brought by green products. In this situation, no alienation exists between individuals and green consumption. Instead, consumers will think that green consumption is their responsibility, and that feelings of powerlessness and meaninglessness will decrease. Compared with consumers who are not self-affirmed, consumers who are self-affirmed have a weaker sense of alienation when faced with conflicting social norm information. Based on the mediating role of alienation in the impact of social norm conflict on green consumption, this study proposes the following hypotheses:

H3: Self-affirmation moderates the mediation of alienation between social norm conflict and green consumption.
H3a: Self-affirmation moderates the mediation of powerlessness between social norm conflict and green consumption.
H3b: Self-affirmation moderates the mediation of meaninglessness between social norm conflict and green consumption.

**FIGURE 1:**
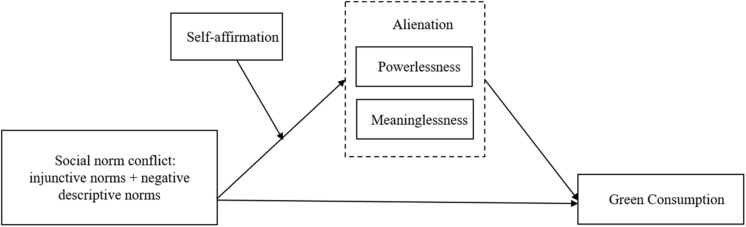
The conceptual model of this study.

Based on the above theoretical review and hypothetical deduction, this study believes that social norm conflict will weaken the role of injunctive norms in promoting green consumption. Alienation (powerlessness and meaninglessness) mediates the weakening effect of social norm conflict on green consumption. Self-affirmation moderates the mediating role of alienation (powerlessness and meaninglessness), whereas social norm conflict influences green consumption. Self-affirmation will reduce the alienation caused by social norm conflict and thus alleviate the weakening influence of social norm conflict on green consumption. The conceptual model of this study is shown in Figure 1.

## Experiment 1

Experiment 1 verified the weakening impact of social norm conflict on green consumption. The study had a three-cell between-subjects design (social norms: injunctive norm vs. negative descriptive norm vs. social norm conflict).

### Participants and Procedure

A total of 141 undergraduates (*M*_age_ = 21.16 years, 74 males) studying at a university in east China participated in the experiment. Participants were randomly assigned to one of three social norms to reduce experimental errors and then asked to read a specific scenario to assess the target social norms. The three scenarios were injunctive norm, negative descriptive norm, and social norm conflict. The detailed instructions they were given are outlined below (adopted from Schultz et al., 2008; White and Simpson, 2013; Wang et al., 2016; Hassell and Wyler, 2018). Simultaneously, key parts of the material are highlighted, and identification questions are compiled based on the material content to enhance participants’ impression of the material. Before reading the social norm material, participants were asked to report their biospheric value (1 = extremely unimportant, 5 = extremely important): preventing pollution; conserving natural resources; respecting the earth, harmony with other species; unity with nature, filling into nature; protecting the environment; and preserving nature (α = 0.83).

#### Injunctive Norm Condition

The results of the green product market survey conducted by the Chinese Consumers Association show that 75% of consumers approve the purchase of green products. In a random interview with one of the consumers, they said “Now the environment is polluted, the ecosystem is degraded, and the environment is the home of human survival, each one of us should protect the environment. It is commendable to take an active part in environmental protection. Buying green products is a very simple and effective way.”

#### Negative Descriptive Norm

The results of the green product market survey conducted by the Chinese Consumers Association show that 75% of consumers do not actively buy green products.

#### Social Norm Conflict Condition

The material of social norm conflict is the combination of an injunctive and negative descriptive norm. Simultaneously, the key parts of the material are highlighted.

After reading the social norm material, participants completed manipulation checks for message appeal. Here, we used a 7-point Likert scale (White and Simpson, 2013), including injunctive norm (“The results you viewed asked you to consider what others want you to do”) and negative descriptive norm (“The results you viewed asked you to consider what others are not doing”). Next, the situation simulation was used to measure the green consumption intention of the participants. Green products refer to all products that can improve environmental quality (Seuring and Müller, 2008). Green is just a relative concept. Based on the above considerations, this experiment uses environmentally friendly water cups as specific experimental objects (Sheng and Ge, 2019). Referring to the green product experimental materials used in the research by Ku et al. (2012), the research team invented “A water cup” and “B water cup” of the same brand for the two products, and it is emphasized that the shape, capacity, and thermal insulation effect of the two water cups are the same. The two water cups are different only in the degree of environmental hazards produced in the production process. The product information of A cup emphasizes functional features, whereas that of B cup emphasizes environmental features. Simultaneously, considering the price difference between green products and ordinary products, we set the price of green products as relatively high rather than very high, as the price of green products is generally 20–25% higher than ordinary ones (Lin and Chen, 2016). The price of cup A is set at 60 yuan, and the price of cup B is set at 75 yuan. After reading the product information of the water cup, we asked the participants to evaluate the relative greenness of the two water cups. Based on a 7-point scale, 1 is the assigned value for cup A (means “A is more environmentally friendly”), whereas 7 is the assigned value for cup B (means “B is more environmentally friendly”). Then, participants were instructed to imagine that they need to purchase a water cup because of their daily needs and let them make a relative choice between cup A and cup B, using a 7-point scale: 1 means “willing to buy cup A,” and 7 means “willing to buy cup B” (Griskevicius et al., 2010). Finally, participants completed the items on demographic information.

### Results and Discussion

#### Manipulation Checks

Biospheric values did not significantly differ among the three groups: *F*(2,138) = 0.530, *p* = 0.590. The one-way ANOVA results showed that an injunctive norm check revealed a main effect for material appeal [*F*(2,138) = 192.229, *p* = 0.000]. Those in the injunctive condition viewed the appeal as more injunctive (*M* = 6.10, *SD* = 0.85) than those in the negative descriptive condition (*M* = 2.43, *SD* = 1.15) and not than those in the conflict condition (*M* = 5.7, *SD* = 1.01). A similar analysis of the negative descriptive norm check also revealed a main effect for material appeal [*F*(2,138) = 194.643, *p* = 0.000]. Those in the negative descriptive condition viewed the appeal as being more negative descriptive (*M* = 5.72, *SD* = 1.14) than those in the injunctive condition (*M* = 2.30, *SD* = 0.85) and not than those in the conflict condition (*M* = 5.95, *SD* = 1.18). These findings suggest that the manipulation was successful.

Since the green degree was measured by a single 7-point scale, the scale score was compared with the median value of 4. The data show that the average value of greenness is 6.34 (*SD* = 0.85), and single sample *t*-test results indicate that the greenness degree is significantly greater than 4. The results of the manipulation check reveal a significant effect of the greenness of the product: *t*(141) = 36.389, *p* < 0.001.

#### Main Effect Analysis

One-way ANOVA results showed that there were significant differences in green consumption intention among the three groups, *F*(2,138) = 70.845, *p* = 0.000. *Post hoc* analysis revealed that participants in the negative descriptive norm condition were less willing to engage in green consumption than those in the injunctive norm condition (*M*_negative descriptive_ = 3.02, *SD* = 0.85; *M*_injunctive_ = 5.47, *SD* = 0.89; negative descriptive norm vs. injunctive norm: 95% confidence interval (CI) for mean difference [−2.93, −1.97], *p* = 0.000, Table 1). Meanwhile, the green consumption willingness of the social norm conflict condition (injunctive norms + negative descriptive norms) was significantly lower than that of the injunctive norm condition (*M*_norm conflict_ = 4.55, *SD* = 1.02; *M*_injunctive_ = 5.47, *SD* = 0.89; social norm conflict vs. injunctive norm: 95% CI for mean difference [−2.00, −1.05], *p* = 0.000). These findings prove that negative descriptive norm weakens the role of injunctive norm in green consumption. Thus, the results support H1.

**TABLE 1:** Multiple comparisons of green consumption of three conditions.

Conditions		I–J	*SE*	*p*	95% CI	
					LLCI	ULCI
Injunctive norms	Negative descriptive norms	2.45*	0.19	0.000	1.97	2.93
	Social norm conflict	0.92*	0.19	0.000	0.45	1.39
Negative descriptive norms	Injunctive norms	−2.45*	0.19	0.000	−2.93	−1.97
	Social norm conflict	−1.52*	0.19	0.000	−2.00	−1.05
Social norm conflict	Injunctive norms	−0.92*	0.19	0.000	−1.39	−0.45
	Negative descriptive norms	1.33*	0.19	0.000	1.05	2.00

*p < 0.05.

#### Discussion

The green consumption willingness of the negative descriptive norm is significantly lower than that of the injunctive norm condition, and the green consumption willingness of the social norm conflict condition (injunctive norms + negative descriptive norms) is significantly lower than that of the injunctive norm condition. This indicates that the social evaluation of green consumption contained in the injunctive norms encourages consumers to engage in green consumption, whereas the non-green consumption behaviors of others described in the negative descriptive norms do not encourage consumers to engage in green consumption. In the context of the coexistence of injunctive norms and negative descriptive norms, negative descriptive norms weakened the role of injunctive norms in promoting green consumption. In Experiment 2, we further explore the psychological mechanism by which social norm conflict affects green consumption.

## Experiment 2

The purpose of Experiment 2 was to verify the mediating role of alienation in the process of conflicting social norms weakening the role of green consumption. Specifically, it tests the mediating role of powerlessness and meaninglessness in the process of social norm conflict weakening the role of green consumption. The study had a two-cell between-subjects design (social norms: injunctive norm vs. social norm conflict).

### Participants and Procedure

A total of 131 undergraduates (*M*_age_ = 21.71 years, 67 males) participated in the study. They were randomly divided into two groups. All participants were asked to report their biospheric value as in Experiment 1 and were randomly assigned to read a specific scenario of injunctive norms and social norm conflict as in Experiment 1. After reading the social norm material, participants completed manipulation checks same as in Experiment 1 and answered a set of questions using a 7-point Likert scale to measure their powerlessness (α = 0.84), meaninglessness (α = 0.86), and their willingness to engage in green consumption as in Experiment 1. We used 10 items adapted from Mottaz (1981) to measure powerlessness and meaninglessness, such as “The effect of buying green products on the environment is small”; “Buying green products at the present stage is not worth doing”; “I am thinking of the purpose of buying green products”; “It is difficult for me to associate green products with environmental protection”; “I can hardly believe that buying green products has a positive effect on environmental protection”; and “Even if I buy green products, the environmental benefits are minimal.”

### Results and Discussion

#### Manipulation Checks

Biospheric values did not significantly differ among the two groups: *t*(129) = 0.236, *p* = 0.813. Independent sample t-test results showed that those in the injunctive condition viewed the appeal as being not more injunctive (*M* = 5.91, *SD* = 0.94) than those in the conflict condition (*M* = 5.76, *SD* = 0.85), *t* = 0.95, *p* = 0.344. Those in the conflict condition viewed the appeal as being more negative descriptive (*M* = 5.88, *SD* = 1.03) than those in the injunctive condition (*M* = 2.48, *SD* = 1.01), *t* = 19.012, *p* = 0.000. These findings suggest that the manipulation was successful.

#### Main Effect Analysis

To test the weakening effect of social norm conflict on the promotion of green consumption by social norms, an independent sample *t*-test was performed on the willingness to engage in green consumption among the two conditions of participants. The results showed that the green consumption intention of the injunctive norm condition (*M*_injunctive norm_ = 5.43, *SD* = 1.08) was significantly higher than that of the social norm conflict condition (*M*_norm conflict_ = 4.43, *SD* = 0.97), *t* = 5.51, *p* < 0.001, thereby proving H1 once more.

#### Mediating Effect Analysis

**FIGURE 2:**
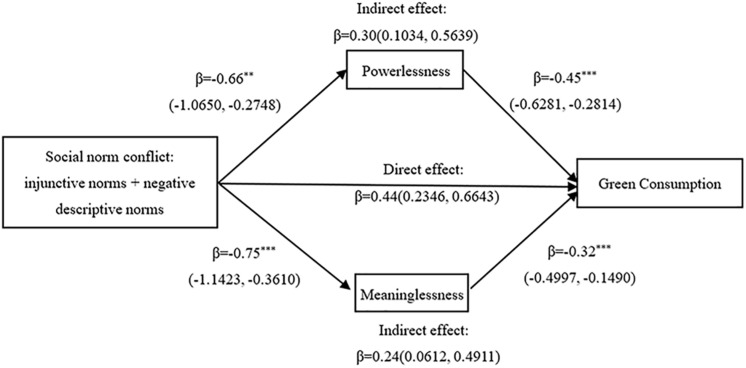
The mediating effect of alienation. ^∗∗^*p* < 0.01, ^∗∗∗^*p* < 0.001.

We tested the mediating role of powerlessness and meaninglessness in the weakening effect of social norm conflict on injunctive norms promoting green consumption. We implemented a bootstrapping analysis that generated a sample size of 5,000 (Hayes, 2013; Model 4) to test the mediating role of powerlessness and meaninglessness between social norm conflict and green consumption. The independent variable was social norms (injunctive norm, 1; social norm conflict, 0). The result demonstrates that the 95% CI for the indirect effect of powerlessness was significant and excluded 0 (β = 0.30, 95% CI: [0.1034,0.5639]), which means that powerlessness plays a mediating role between social norm conflict and green consumption, as shown in Figure 2. In addition, the 95% CI for the indirect effect of meaningless was significant and excluded 0 (β = 0.24, 95% CI: [0.0612, 0.4911]). Therefore, hypothesis H2a and hypothesis H2b are supported. Thus, it is assumed that H2 is also supported.

#### Discussion

Experiment 2 tested the mediating role of alienation in the process of social norm conflict affects green consumption. Specifically, powerlessness and meaninglessness play a mediating role in the relationship between the two. In the next experiment, we further explore how to solve the weakening role of social norm conflict in green consumption.

## Experiment 3

Experiment 3 verified the moderating effect of self-affirmation on the mediating effect of alienation, specifically to verify that the mediating relationship between powerlessness and meaninglessness in social norm conflict and green consumption is regulated by self-affirmation, namely, to verify hypothesis H3a and hypothesis H3b.

### Participant and Procedure

This experiment needs to elicit self-affirmation and target social norms. For the order of manipulation of social norms and self-affirmation, the timing of self-affirmation may affect the degree of processing of social norm information. Most studies show that manipulating self-affirmation at the beginning of the experiment or after the subject is exposed to self-threat information will effectively reduce the defensive processing of the subject to self-threat information, but the premise is that it is effective only when the participant has no chance to react defensively after facing self-threatening information. In other words, self-affirmation cannot relieve the defensive response that has already occurred. Moreover, the timing of self-affirmation also affects the acceptance of the persuasive information by the participants (He and Huang, 2012). Since self-affirmation can increase participants’ self-confidence, they will be less disturbed by persuasive information (Brinol et al., 2007). Subjects’ self-affirmation before and after receiving persuasive information may produce opposite results. According to the self-efficacy hypothesis, after receiving the persuasive information, individuals form the first level of idea, they will rethink the idea, the individual’s attitude toward persuasive information does not affect the behavior, and they are also affected by the confidence level of the individual in persuasive information (Petty et al., 2007). If before receiving the persuasive information self-affirmation is carried out, since self-affirmation will increase an individual’s self-confidence, the individual will reduce the motivation for careful handling of persuasion information, and the persuasion effect will be weakened; when self-affirmation is conducted after receiving the persuasive information, the increased confidence of individuals will increase the first ideas they hold on the persuasive information, reduce the corresponding thinking, and trust their judgment more, thus enhancing the effect of persuasion. Based on the above considerations, for the order of manipulation of social norms and self-affirmation, this study first manipulates the subject’s self-affirmation and then manipulates social norms.

The study was a 2 (social norms: injunctive norm vs. social norm conflict) × 2 (self-affirmation: high vs. control) between-subjects design. A total of 248 participants completed the study. They were randomly divided into four groups, including 122 males and 126 females, with an average age of 30.47 years old. The study was divided into four parts. First, all participants were asked to report their biospheric value same as in Experiment 1 and completed a state measure of mood using single items (Brief Mood Introspection Scale; Mayer and Gaschke, 1988) to control the participants’ existing self-affirmation level (1 = not at all, 5 = extremely): happy, calm, active, nervous, tired, and sad. Second, the subjects were told to complete two unrelated tasks. One task was a description of a person’s characteristics, which was the manipulation of self-affirmation, by allowing the subjects to affirm the core value of the individual (Napper et al., 2009; Cambon and Yzerbyt, 2018). The selection of core values is based on the questionnaire compiled by Cloninger (2005), which reflects the six major virtues in human nature (wisdom, courage, love, justice, temperance, and excellence). The six major virtues contained a total of 24 positive psychological qualities, from which 16 excellent qualities were selected. Specifically, the participants were asked to score the performance of the 16 excellent qualities listed in the questionnaire using a 7-point Likert scale and choosing one item that they thought best reflected their own best advantages. Afterward, write the reasons and give examples in daily life. Participants without self-affirmation must choose their favorite fruit from the 16 listed in the questionnaire and write the reason. This method has been widely used in previous studies to manipulate self-construal (Napper et al., 2009). After completing the above tasks, subjects answered four questions to test the results of self-affirmation manipulation, such as “the above writing task will make me think about the positive aspects of myself,” and adopted a 7-point Likert scale. Another task is in manipulating social norms. The manipulation of social norms is same as in Experiment 1, that is, let the subjects read the corresponding material of the injunctive norms and social norm conflict. Participants completed social norms manipulation checks same as in Experiment 1.

The third part is to measure powerlessness (α = 0.86) and meaninglessness (α = 0.89). The measurement of powerlessness and meaninglessness is the same as in Experiment 2. The fourth part is the measurement of green consumption willingness. After collective discussion by the research team, the environmental protection notebook was selected as the specific green product. Referring to the specifications of the green product experimental materials used in the research by Ku et al. (2012), two products of the same brand “A notepad” and “B notepad” were presented. It is emphasized that the specifications of these two notepads are the same. The difference between the two notepads is in the raw materials. The product information of A notepad emphasizes the functional characteristics: “the production of high-density Dowling paper, fluent writing, clear handwriting, tight and opaque texture, non-bleeding, page-turning resistance, easy damage, good water resistance, and non-glare paper.” The product information of B notepad emphasizes environmental protection characteristics, particularly “made of high-quality recycled paper, thick and easy to write without ink seepage, yellowish color for eye protection, FSC environmental protection forest system certification, effectively reducing wood consumption, reducing energy consumption, and very environmentally friendly.” Moreover, considering the price difference between green products and ordinary products, to control the price of green products relatively high rather than absolute high, the price of A notepad is set to 10 yuan, and the price of B notepad is set to 13 yuan. Participants were also asked to evaluate the degree of the greenness of the two notepads. Next, participants were instructed to imagine that they need to buy a notepad because of their daily needs and let them make a relative choice between A notepad and B notepad, using a 7-point scale: 1 means “willing to buy A notepad,” and 7 means “willing to buy B notepad.”

### Results and Discussion

#### Manipulation Checks

Biospheric values did not significantly differ among the four groups: *F* = 1.121, *p* = 0.341. Through an average of the sum of mood scores, it did not significantly differ among the four groups, *F* = 0.809, *p* = 0.490. Independent sample *t*-test results showed that those in the injunctive condition viewed the appeal as being not more injunctive (*M* = 5.89, *SD* = 0.86) than those in the conflict condition (*M* = 5.81, *SD* = 0.87), *t* = 0.772, *p* = 0.441. Those in the conflict condition viewed the appeal as being more negative descriptive (*M* = 5.73, *SD* = 1.17) than those in the injunctive condition (*M* = 2.46, *SD* = 0.91), *t* = 24.525, *p* = 0.000. For self-affirmation, participants in the self-affirmed group (*M* = 5.14, *SD* = 0.91) showed more positive thinking than those in the non-self-affirmed group (*M* = 3.99, *SD* = 0.94), *t* = 9.813, *p* = 0.000. These findings suggest that the manipulation of social norms and self-affirmation was successful. As expected, the average value of the green degree is 6.39, which is significantly greater than 4, *t* = 40.121, *p* < 0.001.

#### Moderated Mediating Effect Analysis

Moderated mediating effect analysis tests the moderating effect of self-affirmation on the mediating role of powerlessness and meaninglessness in social norm conflict weakening green consumption. We implemented a bootstrapping analysis that generated a sample with a size of 5,000 (Hayes, 2013; Model 7) to test the moderated mediating role of self-affirmation, and the results are shown in Table 2.

**TABLE 2:** Moderation of self-affirmation on the mediating effect of powerlessness and meaninglessness.

Type	Self-affirmation	Effective value	*SE*	Include/exclude	95% CI	
					LLCI	ULCI
Powerlessness	Yes	−0.14	0.12	Included 0	−0.35	0.09
	No	−0.47	0.16	Excluded 0	−0.83	−0.19
Meaninglessness	Yes	−0.05	0.04	Included 0	−0.18	0.07
	No	−0.27	0.11	Excluded 0	−0.52	−0.09

The spotlight analysis revealed that the 95% CI for the mediating role of powerlessness was not significant and included 0 (β = −0.14, 95% CI: [−0.35, 0.09]) under the self-affirmation condition; the mediating role of powerlessness was significant and excluded 0 (β = −0.47, 95% CI: [−0.83, −0.19]) under the no self-affirmation condition. Thus, the mediating role of powerlessness in the process of social norm conflict weakening green consumption is moderated by self-affirmation. The spotlight analysis revealed that the 95% CI for the mediating role of meaninglessness was not significant and included 0 (β = −0.05, 95% CI: [−0.18, 0.07]) under the self-affirmation condition; the mediating role of meaninglessness was significant and excluded 0 (β = −0.27, 95% CI: [−0.52, −0.09]) under the no self-affirmation condition. Thus, the mediating role of meaninglessness during social norm conflict weakening green consumption is moderated by self-affirmation. Therefore, hypothesis H3a and hypothesis H3b are supported, and, consequently, H3 is supported.

#### Discussion

Experiment 3 tested the moderating effect of self-affirmation on the mediating effect of powerlessness (H3a) and meaninglessness (H3b) in social norm conflict and green consumption.

## Discussion and Conclusion

This study explores the weakening impact of social norm conflicts on green consumption, and, simultaneously, the mechanism of action and how to alleviate the impact of weakening are discussed. First, social norm conflict weakens the role of injunctive norms in promoting green consumption, and, particularly, negative descriptive norms weaken the role of injunctive norms in promoting green consumption. The negative descriptive norms describe that most members of the group are not engaged in green consumption. Currently, the injunctive norms guaranteed by social sanctions do not promote the green consumption behavior of individuals, and the negative descriptive norms weaken the promotion of the imperative norms (Keizer et al., 2008).

Second, alienation (powerlessness and meaninglessness) mediates the weakening effect of social norm conflict on green consumption. Social norm conflicts make consumers believe that their green consumption will not bring about significant environmental improvements and create a “feeling of powerlessness” when others “do nothing.” Moreover, these conflicts will also make consumers become unable to value the effectiveness of green consumption for environmental improvement and understand the actual meaning of green consumption, and this creates a “feeling of meaninglessness,” which reduces their inclination toward green consumption.

Third, the mediating role of alienation in weakening the influence of green consumption by social norm conflict (powerlessness and meaninglessness) is moderated by self-affirmation. Self-affirmation will reduce the alienation caused by social norm conflict, thus alleviating the weakening influence of social norm conflict on green consumption. Self-affirmed consumers think more about their own advantages and positive aspects, maintain their complete self-perfection, and have high moral integrity (Sherman and Cohen, 2006; Lv and Zhao, 2017). They can calmly process the social norm conflict information of green consumption. Currently, no psychological alienation between consumers and green consumption exists, and this alleviates the weakening impact of social norm conflicts on green consumption.

### Implication

This paper has three main contributions to the field. First, reduce the activation level of negative descriptive norms for green consumption and increase the activation level of positive descriptive norms for green consumption. To solve the problem of social norm conflict of green consumption, reducing the activation level of negative descriptive norms of green consumption, so that they do not conflict with injunctive norms, is vital. The government should reduce the implementation cost of green consumption behavior through fiscal subsidies, tax incentives, and consumption rewarding points. The government should make green consumption a positive descriptive norm by increasing the supply of green products, improving the community resource recycling system, and building a sharing economy.

Second, the government should enhance the role of injunctive norms in monitoring and promoting green consumption and preventing probable social norm conflict situations. When green consumption has not yet become a consumer’s conscious behavior, the government should use injunctive norms to keep the green consumption high-pressure situations of the social atmosphere through media publicity, policy guide, and so on. The government should increase the exposure and criticism of non-green consumption, reduce the negative descriptive activation level of consumers, and then guide the formation of green consumption.

Third, during green consumption marketing, self-affirmation should be used purposely to reduce the alienation between consumers and green consumption. The existing marketing information of green products is mostly presented in the form of an information framework. This information presentation method will, to a certain extent, make consumers habitually resist. In the product marketing process, adopting self-affirmation methods, for example, to affirm the simple environmental behaviors consumers have done before, such as garbage disposal, turning off lights on time, and so on. This will improve overall self-cognition, reduce the consumer’s resistance to green consumption, and allow the consumer to be more objectively aware of green consumption and make green consumption decisions.

### Limitations

This research analyzes the relationship between social norm conflicts and green consumption, but some limitations and directions remain for future research. First, all research was conducted in the laboratory, which, to some extent, limited their externality. The measurement of green consumption in this study does not reflect the actual purchase behavior of consumers, but it is the green consumption tendency of consumers when compared with ordinary products. Future research should be conducted in real consumption scenarios to minimize experimental deviation. Second, the present research controls participants’ level of existing self-affirmation level by measuring mood, and results show that it can be controlled. But the measurement of mood could reflect the situational self-affirmation level to some extent, and the characteristic self-affirmation was not measured and controlled. The present research controls participants’ level of social norms, existing preference for green products, and other variables by random grouping, but existing ideas about the social norms, preference for green products, and other variables were not measured and controlled. Future research should consider the measurement of existing ideas about the social norms and the characteristic self-affirmation level to minimize experimental deviation. Third, additional mediating or moderating variables may exist that can explain and alleviate the weakening impact of the social norm conflict on green consumption. Therefore, additional possible mediating and moderating variables should be included to explore the influence of the social norm conflict on green consumption.

## Data Availability Statement

The raw data supporting the conclusions of this article will be made available by the authors, without undue reservation.

## Ethics Statement

The studies involving human participants were reviewed and approved by Qilu University of Technology (Shandong Academy of Sciences) Research Ethics Committee. The patients/participants provided their written informed consent to participate in this study.

## Author Contributions

WG and GS involved in all steps of the study. HZ performed the statistical analysis and revised the manuscript. All authors contributed to the article and approved the submitted version.

## Conflict of Interest

The authors declare that the research was conducted in the absence of any commercial or financial relationships that could be construed as a potential conflict of interest.
